# Supplementary material for: The Systematic Workplace-Improvement Needs Generation (SWING): Verifying a Worker-Centred Tool for Identifying Necessary Workplace Improvements in a Nursing Home in Japan
Source: Int J Environ Res Public Health. 2022 Feb 1;19(3):1671. doi: 10.3390/ijerph19031671 (PMC8835352; doi:10.3390/ijerph19031671)
Supplement: Supplementary file 1 [file ijerph-19-01671-s001.zip › Supplement1_SWING_JPN.pdf]

Supplement 1 (Questionnaire S1): SWING (Japanese version)

以下の質問事項について、**順番に回答し、**表に記入をして下さい。

1. **あなたにとって、どのような職場が働きやすい**ですか。
 大切だと思うことを「必ず５つ」挙げてください。
 単語ではなく、文章で具体的に記載して下さい。

2. **いま挙げてもらった５つについて、現在、どの程度、満たされていますか。**
 １００点満点中何点で答えてください。**５つの項目、それぞれに回答**、お願いします。

3. ５つの項目について、**重要性のバランス**をお尋ねします。
 ５つの合計が１００%になるように、それぞれの割合を教えて下さい。

	1. どのような職場が働きやすいか。 大切だと思うこと､必ず5つ挙げて下さい。	2. どの程度満たされているか 項目ごと 100 点満点中	3. 5 つの重要性のバランス 全部の合計が 100%
1		(100 点満点中) 点	% ＋
2		(100 点満点中) 点	% ＋
3		(100 点満点中) 点	% ＋
4		(100 点満点中) 点	% ＋
5		(100 点満点中) 点	% ＝
			**合計** １００ %
