# Supplementary material for: The Systematic Workplace-Improvement Needs Generation (SWING): Verifying a Worker-Centred Tool for Identifying Necessary Workplace Improvements in a Nursing Home in Japan
Source: Int J Environ Res Public Health. 2022 Feb 1;19(3):1671. doi: 10.3390/ijerph19031671 (PMC8835352; doi:10.3390/ijerph19031671)
Supplement: Supplementary file 1 [file ijerph-19-01671-s001.zip › Supplement2_SWING_ENG.pdf]

Supplement 2 (Questionnaire S2):
SWING (English version)

Please answer the following questions (in order) and fill the required information in the table given.

1. **What kind of workplace is easy/comfortable for you to work in?**
   - **<u>Please list five things</u> that you think are important.**
   - **Please be specific in your answer, using sentences instead of words.**

2. **To what extent have these five things been fulfilled?**
   - **How many points (<u>out of 100)</u> would you give to <u>each of the five items?</u>**

3. **How would you rank the five items in order of importance?**
   - **<u>Please state the percentage of each item so that the total of the five items adds up to 100%.</u>**

	**1. What kind of workplace is easy/comfortable for you to work in?**	**2. To what extent have these five things been fulfilled? (out of 100)**	**3. How would you rank the five items in order of importance? (the total of the five items adds up to 100%)**
1		points	% +
2		points	% +
3		points	% +
4		points	% +
5		points	% =
			**<u>Total</u>** **<u>100 %</u>**
