# Supplementary material for: The Systematic Workplace-Improvement Needs Generation (SWING): Verifying a Worker-Centred Tool for Identifying Necessary Workplace Improvements in a Nursing Home in Japan
Source: Int J Environ Res Public Health. 2022 Feb 1;19(3):1671. doi: 10.3390/ijerph19031671 (PMC8835352; doi:10.3390/ijerph19031671)
Supplement: Supplementary file 1 [file ijerph-19-01671-s001.zip › Supplement3_distribution.pdf]

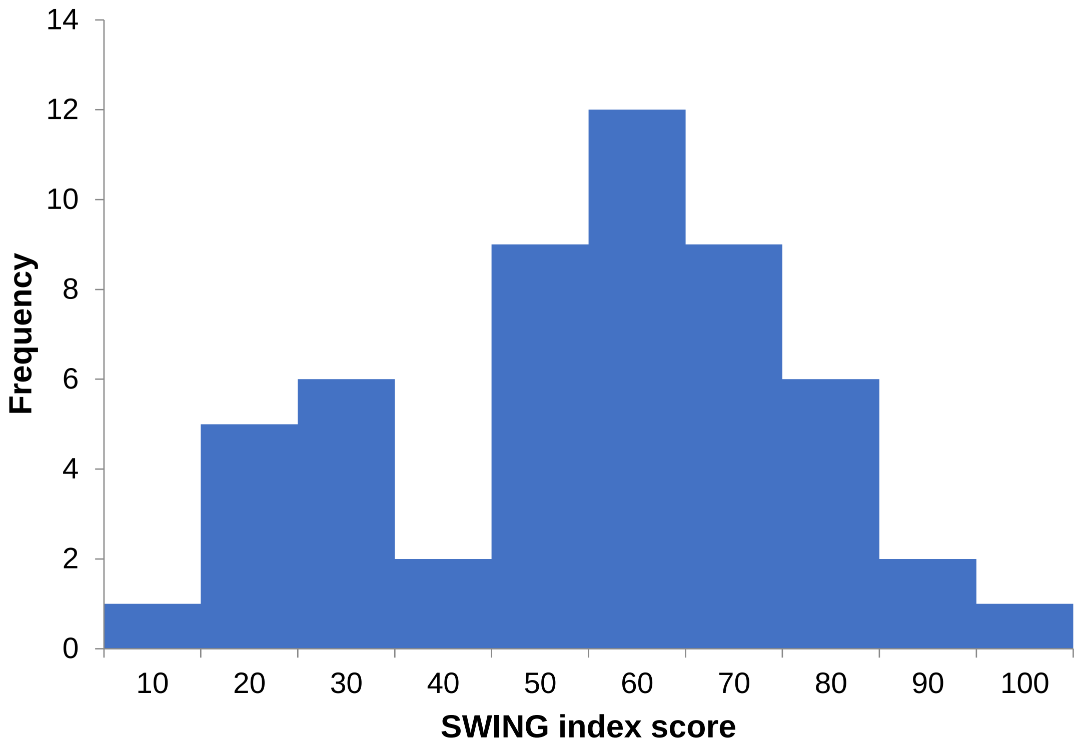

Supplement 3 (Figure S1). Distribution of SWING index score (mean=51.5, SD=21.2). The normality of the distribution was confirmed by Shapiro-Wilk test ($p = 0.145$).
